# Long-lived lung megakaryocytes contribute to platelet recovery in thrombocytopenia models

**DOI:** 10.1172/JCI181111

**Published:** 2024-09-19

**Authors:** Alison C. Livada, Kathleen E. McGrath, Michael W. Malloy, Chen Li, Sara K. Ture, Paul D. Kingsley, Anne D. Koniski, Leah A. Vit, Katherine E. Nolan, Deanne Mickelsen, Grace E. Monette, Preeti Maurya, James Palis, Craig N. Morrell

**Affiliations:** 1 Aab Cardiovascular Research Institute,; 2 Department of Pathology and Laboratory Medicine,; 3 Department of Pediatrics,; 4 Department of Comparative Medicine,; 5 Department of Microbiology and Immunology, and; 6 Department of Medicine, University of Rochester School of Medicine and Dentistry, Rochester, New York, USA.

**Keywords:** Hematology, Platelets

## Abstract

Lung megakaryocytes (Mks) are largely extravascular with an immune phenotype (1). Because bone marrow (BM) Mks are short lived, it has been assumed that extravascular lung Mks are constantly “seeded” from the BM. To investigate lung Mk origins and how origin affects their functions, we developed methods to specifically label lung Mks using CFSE dye and biotin delivered via the oropharyngeal route. Labeled lung Mks were present for up to 4 months, while BM Mks had a lifespan of less than 1 week. In a parabiosis model, lung Mks were partially replaced over 1 month from a circulating source. Unlike tissue-resident macrophages, using MDS1^-Cre-ERT2^ TdTomato mice, we found that lung Mks arose from hematopoietic stem cells. However, studies with FlkSwitch mTmG mice showed that lung Mks were derived from a Flt3-independent lineage that did not go through a multipotent progenitor. CFSE labeling to track lung Mk–derived platelets showed that approximately 10% of circulating platelets were derived from lung-resident Mks at steady state, but in sterile thrombocytopenia this was doubled (~20%). Lung-derived platelets were similarly increased in a malaria infection model (*Plasmodium yoelii*) typified by thrombocytopenia. These studies indicate that lung Mks arise from a Flt3^–^ BM source, are long-lived, and contribute more platelets during thrombocytopenia.

## Abstract 2

Extravascular lung megakaryocytes are long-lived cells that produce more platelets in thrombocytopenia

## Graphical abstract

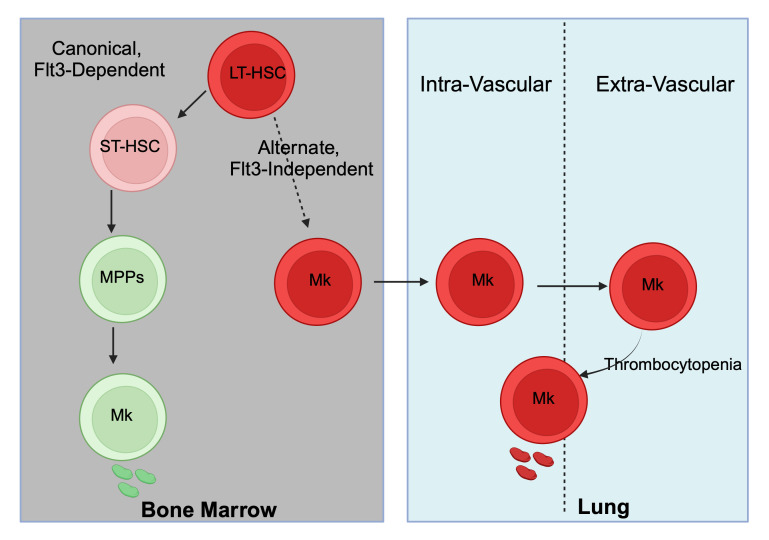

## Introduction

Platelets serve dual roles as a critical component of clot formation and as regulators of immune responses (1, 2). Activated platelets promote inflammation, while circulating resting platelets limit inflammation and maintain vascular permeability (3–6). Bone marrow (BM) megakaryocytes (Mks) serve as the primary producers of circulating platelets (7, 8), although the presence of Mks in the lung has been described for more than a century (9–11). Recent endeavors to characterize lung Mks have begun to define their unique phenotype (12) and demonstrated that lung Mks contribute to platelet production (13), as well as modulate local immune responses (14, 15). A clearer definition of the functions and origins of lung Mks will expand our knowledge of Mk roles in vascular biology and provide a better understanding of platelet heterogeneity in health and disease.

While Mks in the lung make platelets, their relative contribution to the total platelet pool remains controversial, with estimates ranging from 7% to 50% (10, 13, 16). Lefrancais, Ortiz-Muñoz, and co-authors estimated the number of platelet fragments generated per lung Mk over time using 2-photon intravital microscopy, and by scaling up these measurements to the full lung volume, they estimated that lung Mks make up 50% of the platelet pool (13). Others have used different imaging methods to extrapolate platelet production potential and proposed that lung Mks contribute fewer numbers of platelets (8). To date, only a few studies have compared lung and BM Mk phenotypes and have indicated that the roles and functions of lung Mks may be quite different from those of BM Mks. In addition to platelet production, Mks in the lung have an immune-modulatory phenotype (15, 17) and can act as antigen-presenting cells (17). The lifespan of lung Mks has not, to our knowledge, been studied, although they are assumed to have a life cycle similar to that of BM Mks despite the fact that the majority of lung Mks are extravascular and 2n. Based on primarily in vitro and some in vivo data, the life cycle of BM Mks includes maturation, polyploidization, and platelet production, which requires approximately 5–7 days in humans and 2–3 days in rodents, after which they have exhausted their capacity to produce platelets (18, 19). Lung Mks are largely 2N, and their turnover during homeostasis and pathological states is not known. Additionally, whether lung Mk immune function confers vulnerability, resistance, or heightened responses to physiologic challenges has not been studied, to our knowledge.

The origin of lung Mks is also largely undefined. Lung Mks are assumed to arise from a BM Mk progenitor (MkP) or from mature Mks that leave the BM and travel in the circulation to seed the lung (12, 20). Most reports on BM megakaryopoiesis describe BM hematopoietic stem cells (HSCs) giving rise to BM Mks in a differentiation process that occurs through successive progenitors with diminishing lineage capacity, until arriving at the MkPs (19, 21–23). There is also a less defined “direct” pathway from HSC to MkP or Mk (24–28) that may be particularly salient during immune challenge (26). The lineage of lung Mks and whether they arise from successive progenitors or directly from HSCs either locally or from the BM is not known.

We now demonstrate that lung Mks are long-lived cells that comprise approximately 10% of the circulating platelet pool at steady state and increase their relative platelet production in the setting of thrombocytopenia. Using lineage-tracing reporter mice, we found that lung Mks are an HSC-dependent population but arise via a Flt3-independent lineage from HSCs, in contrast to largely Flt3-dependent differentiation for BM Mks. These data indicate a distinct lineage for lung Mks and suggest that the Mk origin may confer lung Mks with a longer lifespan and greater contribution during low platelet states. These data functionally indicate that resident lung Mks are a stable source of platelets in times of increased demand.

## Results

### Lung Mks are long-lived cells compared with BM Mks.

**Figure 1:**
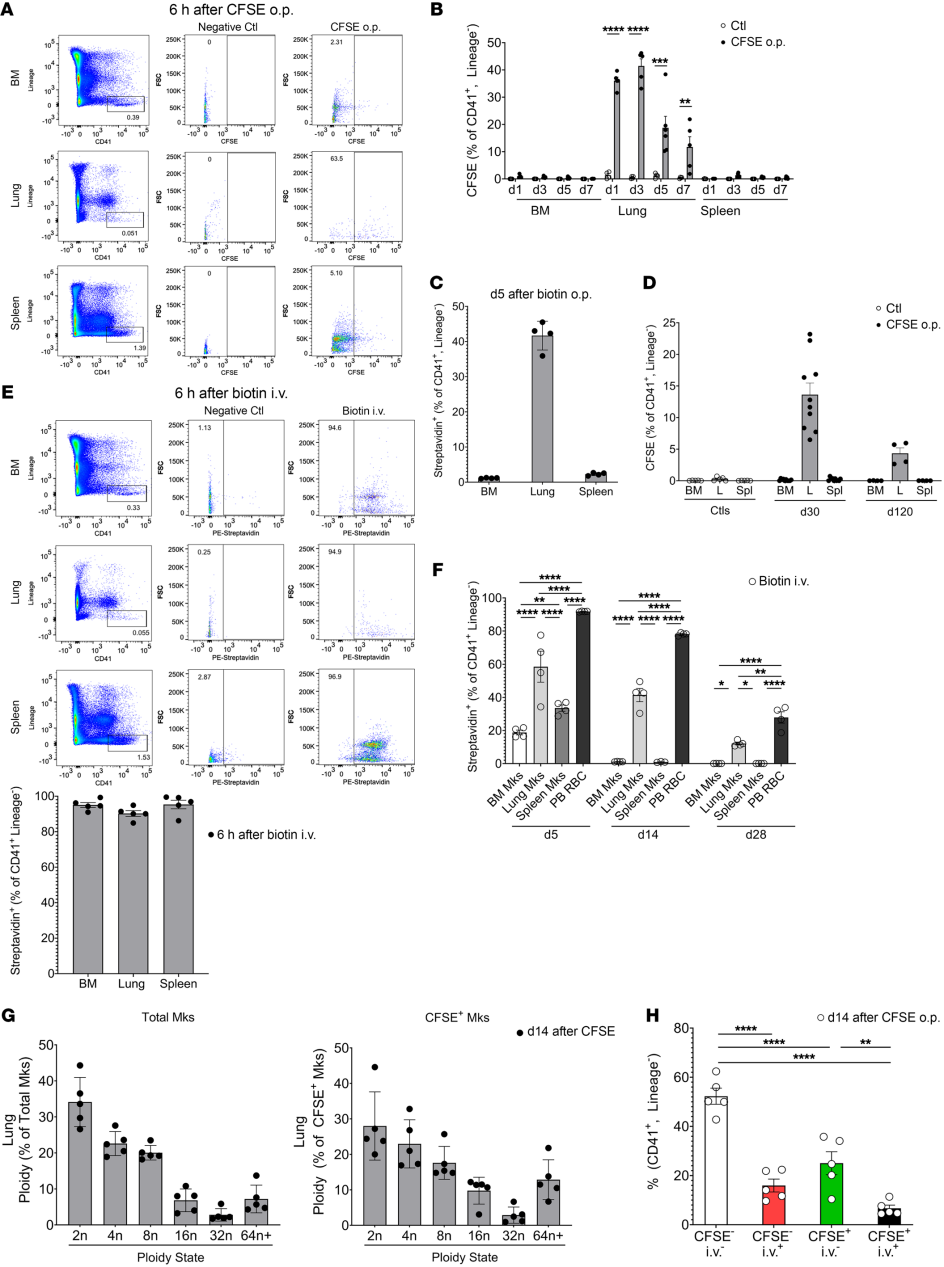
Lung Mks are long-lived cells. (**A** and **B**) CFSE or (**C**) biotin was delivered o.p. to mice and showed specific labeling of lung Mks, but not BM or splenic Mks. (**A**) CFSE 6 hours later (*n* = 4, 2 independent experiments), (**B**) CFSE on day 1 (d1) to day 7 (d7) (*n* = 4–5 per group from 2 independent experiments) and (**C**) on day 5 after biotin o.p. labeling (*n* = 4; results are representative of 2 independent experiments). (**D**) CFSE-labeled lung Mks were present up to 120 days after CFSE administration (*n* = 10 from 2 independent experiments for day 30; *n* = 4 for day 120 from 1 independent experiment). L, lung; Spl, spleen. (**E**) Representative flow cytometry and quantitation results 6 hours after biotin i.v. labeling of BM, splenic, and lung Mks, similar to **A** (*n* = 4; results are representative of 2 independent experiments). (**F**) Biotin-labeled lung but not BM or splenic Mks were present up to 28 days following i.v. delivery (*n* = 4; results are representative of 2 independent experiments). RBCs were used as a positive control. PB, peripheral blood. (**G**) Total lung Mks and CFSE^+^ Mks had similar ploidy (day 14 after CFSE) (*n* = 5, representative results are from 2 independent experiments). (**H**) CFSE^–^ and CFSE^+^ lung Mks had a similar intravascular (CD42d^+^) and extravascular (CD42d^–^) distribution (CD42d given i.v.) (*n* = 5; results are representative of 2 independent experiments). Data indicate the mean ± SEM. *P < 0.05, **P < 0.01, ***P < 0.001, and ****P < 0.0001, by (**B**) multiple *t* tests with Holm-Šidák multiple-comparison correction, (**D** and **F**) 2-way ANOVA with Tukey’s multiple-comparison correction, and (**H**) 1-way ANOVA with Šidák multiple-comparison correction. Ctl, control; FSC, forward scatter.

To begin to investigate the function and lifespan of lung Mks, we modified protocols used to track immune cells (29, 30) and developed a new strategy to label lung but not BM Mks. We administered CFSE dye, a cell-permeable, fixable dye that allows for long-term and stable cell labeling, via the oropharyngeal (o.p.) route; the dye labeled lung cells, but not cells in other tissue beds (Figure 1A). Six hours after administering CFSE dye o.p. to WT mice, we used flow cytometry to measure CFSE in Mks in BM, lung, and spleen. Mks were identified as CD41^+^ and lineage^–^ (lineage channel included antibodies to exclude the differentiated hematopoietic cells described in Methods and Table 1) to avoid counting other hematopoietic lineages with attached platelets (31). Lung Mks were CFSE^+^ (Figure 1A: middle row), whereas BM (Figure 1A, top row) and spleen (Figure 1A: bottom row) Mks were not. To verify that lung Mks were consistently exposed to CFSE via the o.p. route, we measured labeling 6 hours after CFSE administration, as others have noted that o.p. delivery can expose lung tissues heterogeneously (32). We found that CFSE dye via the o.p. route labeled more than 90% of Mks 6 hours after its administration (Supplement 1A). To determine a time course for lung Mk labeling, we determined the presence of CFSE^+^ Mks on days 1, 3, 5, and 7 after CFSE o.p. administration. We observed negligible numbers of CFSE^+^ Mks in the BM and spleen, whereas a substantial number of CFSE^+^ Mks remained in the lung over the 7-day time frame (Figure 1B). We validated that the CD41^+^lineage^–^ cells identified in the lung were differentiated Mks using CD42d (glycoprotein-V) staining (33), which showed that more than 90% of lung Mks (CD41^+^lineage^–^) were also CD42d^+^ (Supplemental Figure 1B). This method also labeled lineage^–^c-Kit^+^ (LK) cells in the lung, but not in the BM or spleen (Supplemental Figure 1C; supplemental material available online with this article; https://doi.org/10.1172/JCI181111DS1). Therefore, delivery of CFSE o.p. yielded tissue-specific labeling of lung Mks and LKs, with minimal spillover to the BM or spleen. As a complementary approach, we labeled cells with EZ-Link-NHS-biotin (referred to hereafter as biotin label) (34). Biotin was administered o.p. on day 0 to WT mice and streptavidin binding used to identify biotin-labeled cells. On day 5 after o.p. biotin delivery, we found that approximately 40% of lung Mks were streptavidin^+^, whereas no streptavidin binding was detected in BM or splenic Mks (Figure 1C). These data indicated that at least a population of lung Mks lived in the lung for an extended time.

To assess the long-term lifespan of lung Mks, we gave CFSE o.p. to mice and waited until day 30 or day 120 to harvest BM, lungs, and spleens. Surprisingly, a substantial number of lung Mks remained CFSE^+^ on both day 30 and day 120 after labeling, while no CFSE^+^ BM or splenic Mks were detected (Figure 1D). Hematopoietic progenitor LK cells also remained CFSE^+^ only in lung tissue at days 30 and 120 (Supplemental Figure 1D). Because the CFSE dye is not cell lineage specific, we also verified the retention of CFSE dye at day 120 in other cell types that have a long lung residence. B cells (CD20^+^), T cells (CD3^+^), myeloid cells (CD11b^+^), fibroblasts (CD140α^+^), and endothelial cells (CD31^+^) in the lung also retained CFSE dye at day 120 (Supplement Figure 1E). These data imply that at least some lung Mks remained quiescent, long-lived cells or arose from a local CFSE^+^ progenitor.

To provide a comparative analysis of Mk tissue residence, we administered biotin to mice i.v. to label all tissue compartments and waited 6 hours to determine the extent of Mk labeling in the BM, spleen, and lungs. Representative gating demonstrated greater than 90% labeling of Mks in each tissue (Figure 1E, representative gating and quantification). We next determined streptavidin binding–positive Mks on days 5, 14, and 28 after biotin i.p. delivery. Circulating RBCs were used as a positive control (90% labeled on day 5) and, as expected, remained 75% and 30% biotin^+^ on day 14 and day 28, respectively. Approximately 20% of BM Mks remained streptavidin^+^ on day 5, but we detected none after day 5 (Figure 1F). Splenic Mks remained 30% streptavidin^+^ on day 5, but like BM Mks, we detected none at the later time points. In contrast to BM Mks, streptavidin^+^ lung Mks were present at all time points tested (41% and 12% on day 14 and day 28, respectively) (Figure 1F). These data demonstrated that lung Mks had a much longer tissue residence time than did BM Mks.

To further characterize the CFSE^+^ lung Mks, we evaluated their ploidy state and intravascular versus extravascular location on day 14 after CFSE administration. Representative flow cytometric plots of lung and BM Mk ploidy using Hoescht 33342 staining are shown in Supplemental Figure 1F. Similar to our prior report (17), total lung Mks were largely low ploidy (<4n, Figure 1G, left panel). CFSE^+^ Mks were also largely low ploidy with a similar range of ploidy states (Figure 1G, right panel). At this time point, we also found that approximately 25% of total Mks were CFSE^+^ extravascularly and approximately 7% were CFSE^+^ intravascularly (Figure 1H), similar to the overall 3:1 extravascular/intravascular ratio we reported previously (17). Using imaging flow cytometry, in lung Mks from mice treated o.p. with CFSE, we visualized CFSE dye that was not present in lung Mks from control mice (Supplemental Figure 1G). CFSE^+^ Mks were therefore primarily low ploidy and existed in both the extravascular and intravascular space.

Taken together, these labeling studies indicate that at least a subset of lung Mks are long-lived and maintain themselves locally for up to 120 days.

### Lung Mks are only partially replaced by a BM source.

**Figure 2:**
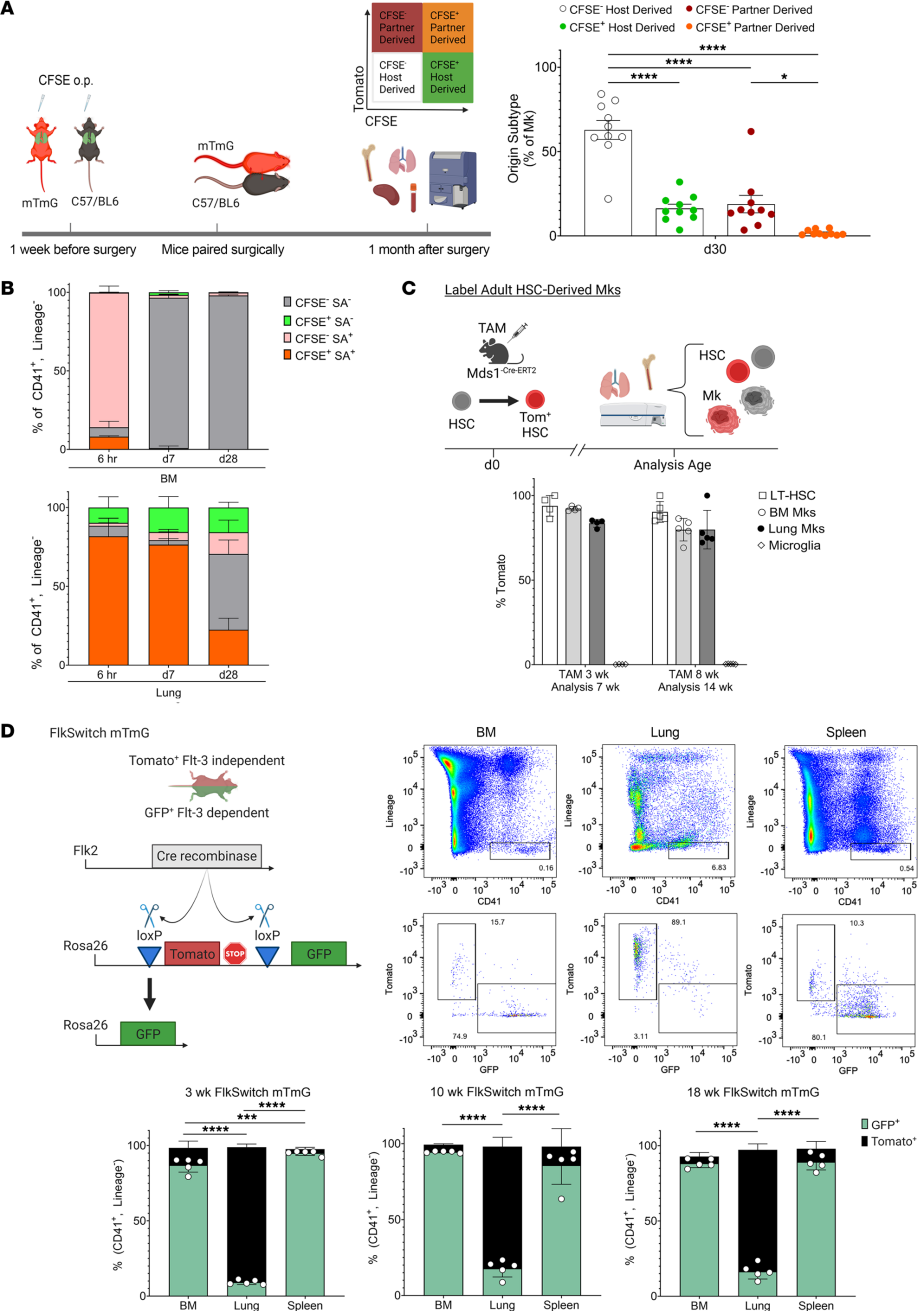
Lung Mks are derived from BM HSCs. (**A**) mTmG and Bl6/J mice were given CFSE o.p., and parabiosis surgeries performed 1 week later. Lung Mks were partially replaced by a BM source (*n* = 8–10 from 2 independent experiments). (**B**) Mice were given CFSE o.p. and biotin i.p., and 6 hours (*n* = 2 from 1 experiment), 7 days (*n* = 3 from 1 experiment), and 28 days (*n* = 5 from 1 experiment) later, BM and lung Mks were assessed. BM Mks were replaced within 7 days, but lung-resident Mks were detected at each time point. (**C**) MDS1-TdTomato^Cre-ERT2^ mice were treated with tamoxifen (TAM) to label HSCs and HSC-derived cells (*n* = 4–5; results are representative of 2 independent experiments). Lung Mks were HSC derived. (**D**) BM, splenic, and lung Mks in FlkSwitch mice were assessed at multiple ages (*n* = 5 per age time point; results are representative of 2 independent experiments) to determine whether Mk differentiation was Flt3 dependent. Lung Mks were largely Tomato^+^Flt3^–^, indicating an HSC-to-Mk differentiation that was distinct from BM and splenic Flt3-dependent differentiation. Data indicate the mean ± SEM. *P < 0.05, ***P < 0.001, and ****P < 0.0001, by (**B**) 1-way ANOVA with Šidák multiple-comparison correction and (**C** and **D**) 2-way ANOVA with Tukey’s multiple-comparison correction and (**A**) 1-way ANOVA with Šidák multiple comparisons correction.

To determine whether long-lived Mks are tissue resident, we used a parabiosis mouse model. WT and mTmG mice (also called ROSAmT/mG) were given CFSE o.p. to label lung cells 1 week prior to parabiosis surgery. Mice were joined for 1 month, after which blood, BM, lungs, and spleens were assessed for parabiont chimerism and CFSE^+^ lung cells (Figure 2A). Alveolar macrophages (AMs) are tissue resident and served as a positive control (35), whereas interstitial macrophages (IMs) are circulation derived and served as a negative control for tissue residence (36). One month after parabiosis surgery, all AMs remained CFSE^+^, host derived (green, Supplemental Figure 2A, left). As expected, the vast majority (77%) of IMs were replaced by CFSE^–^ host-derived cells, and 23% of the IMs were CFSE^–^ partner derived (white, Supplemental Figure 2A, right), indicating circulatory replacement of IMs from the partner parabiont. No IMs were CFSE^+^ from the partner (orange) or host (green). These results demonstrated that parabiosis yielded circulatory mixing and confirmed the CFSE labeling of tissue-resident cells. When we evaluated lung Mks, we found an intermediate phenotype between IMs and AMs (Figure 2B, bottom panel). Like IMs, more than half (63%) of lung MKs were replaced by CFSE^–^ host-derived cells (white) in the 1-month time period. Approximately 19% of lung Mks were CFSE^–^ partner derived (red), with none of the CFSE^+^ cells being partner derived (orange). However, unlike IMs, we noted a subset (~15%–20%) of lung Mks that remained CFSE^+^ host derived (green). These experiments indicated that over a 1-month period, a circulatory source contributed a portion of lung Mks, but also that at least some lung Mks had a longer lifespan and did not undergo circulatory replacement.

To provide a complementary approach and confirm the presence of a subset of lung Mks that have a longer tissue-resident lifespan, we combined the CFSE o.p. and biotin i.p. labeling methods. Mice received CFSE o.p. and biotin i.p. on day 0, and BM and lung Mks were assessed 6 hours later, on day 7, and on day 28 for CFSE dye and biotin labeling. At the 6-hour time point, BM Mks were largely (>90%) CFSE^–^, streptavidin^+^ (Figure 2B, top panel, left, pink bar). On days 7 and 28, BM Mks did not retain biotin labeling and were almost completely double negative (gray bar). The lung was largely double positive at 6 hours (orange bar) and remained so on day 7 (Figure 2B, top panel, right). On day 28, approximately half of the lung was double negative for CFSE and streptavidin, whereas the other half retained either dye, biotin, or both. These results are very similar to the parabiosis model and indicate that a substantial number of lung Mks were tissue resident for over 30 days.

### HSCs give rise to lung Mks largely via a Flt-3–independent pathway.

Given the intriguing discovery of long-lived lung Mks that are replaced by a circulating source, we sought to define the source of lung Mks. In contrast to a circulatory source, lung-resident alveolar macrophages are initially seeded from an HSC-independent developmental source. We therefore leveraged a lineage-tracing model — the MDS1^-Cre-ERT2^ TdTomato mouse model (37) — to track HSC-dependent hematopoiesis. After induction with tamoxifen, TdTomato is expressed in HSCs, and the fluorescent reporter is retained in the subsequent HSC daughter cells (Figure 2C). Tamoxifen administration to mice at 3 weeks of age revealed close to 100% Tomato labeling of BM long-term HSCs (LT-HSCs) collected at 7 weeks (Figure 2C). As a negative control, we used brain microglia, a tissue-resident, HSC-independent cell derived from the yolk sac during embryogenesis (38), and found 0.23% Tomato labeling of microglia. We observed that 90% of BM Mks and 85% of lung Mks were Tomato labeled (Figure 2C). Similarly, when we administered tamoxifen at 8 weeks and collected tissues at 14 weeks, lung and BM Mks were Tomato-labeled while microglia were not (Figure 2C). These data indicated that from the early postnatal period and through adult life, lung Mks are derived from HSCs.

Some Mks arise directly from HSCs, but not through a standard multipotent progenitor (MPP) stage, a process sometimes termed emergency megakaryopoiesis (24, 26, 28, 39). Therefore, to more specifically define the source of HSC-derived lung Mks, we used the FlkSwitch-mTmG model (22), in which cells are Tomato^+^ unless a cell turns on Flt3 protein expression, excises Tomato, and then expresses GFP (Figure 2D). Flt3 is expressed during the transition to short-term HSCs (ST-HSCs) and is highly expressed in MPPs and lymphoid cells (22, 40). Because the recombinase alters the genome, Flt3-expressing progenitors yield daughter cells that remain GFP^+^ (Tomato^+^GFP^+^ represent cells with a recently excised Tomato gene with persistent protein expression; ref. 41). As expected in 3-, 10-, and 18-week-old FlkSwitch-mTmG mice, BM and splenic Mks were largely GFP^+^ (representative gating and quantification, Figure 2D). In contrast, lung Mks were largely (85%–90%) Tomato^+^ (Figure 2D). We also confirmed that expression of Tomato in lung Mks was not due to cleavage of GFP or other tissue preparation–related issues, as lung interstitial macrophages were largely GFP^+^ and alveolar macrophages were Tomato^+^ (Supplemental Figure 2B). We sought to determine whether an immune-differentiated phenotype could be encoded by the Flt3-independent lineage. Therefore, we compared ICAM1 expression between Tomato^+^ and GFP^+^ Mks across tissues, as our prior report showed that ICAM1 was elevated on lung Mks and could be induced on BM Mks following immune stimulation (17). Interestingly, we found that BM Tomato^+^ Mks had increased levels of ICAM1 compared with BM GFP^+^ Mks (Supplemental Figure 2C). Lung Tomato^+^ Mks also had higher levels of ICAM1 than did lung GFP^+^ Mks (Supplemental Figure 2C). However, both Tomato^+^ and GFP^+^ lung Mks had higher levels of ICAM1 than did GFP^+^ BM Mks. We observed no difference in ICAM1 expression between GFP^+^ and Tomato^+^ splenic Mks (Supplemental Figure 2C). These data indicated that the Flt3-independent lineage may have increased the expression of immune markers in the BM and that the lung environment may play an additive role in modifying immune molecule expression. FlkSwitch mice were also given biotin i.p., and 7 days later, streptavidin^+^GFP^+^Tomato^+^ Mks were quantified in the BM, lungs, and spleen. Streptavidin^+^Tomato^+^ Mks remained to a larger extent when compared with streptavidin^+^GFP^+^ Mks in both the lungs and BM (Supplemental Figure 2D), indicating that the prolonged lifespan of Flt3-independent Mks in the lungs may in part be cell intrinsic. Together the FlkSwitch and MDS1 models suggest that lung Mks are derived from HSCs that differentiate largely via a Flt3-independent differentiation pathway.

### Lung-resident, Mk-derived platelets make up a minor portion of the circulating platelet pool at steady state.

**Figure 3:**
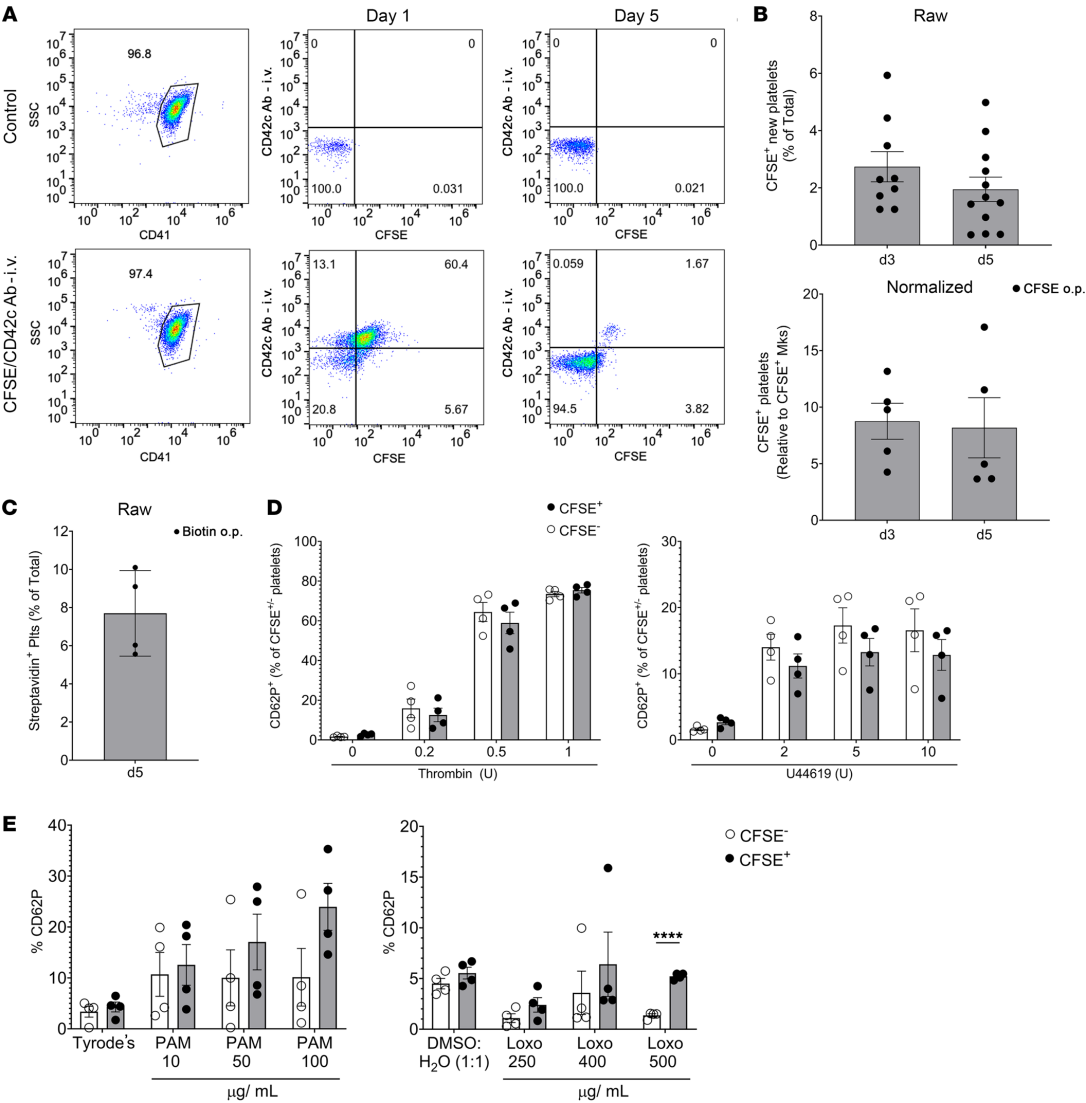
Lung-resident Mks produce platelets. (**A**) Mice were simultaneously given CFSE o.p. and anti-CD42c platelet-labeling antibody i.p. Lung-derived platelets were quantified as CFSE^+^CD42c^–^ platelets by flow cytometry in representative plots. SSC, side scatter. (**B**) Results showed that 2%–5% of total platelets were lung derived (*n* = 9–12 from 3 independent experiments), and when normalized to CFSE^+^ Mks (*n* = 5; results are representative of 2 independent experiments), a maximum of approximately 10% of the platelets were lung-resident, Mk-derived. (**C**) CFSE data were validated by biotin delivered o.p. and by quantification of streptavidin-binding platelets (Plts) (*n* = 4; results are representative of 2 independent experiments). (**D**) CFSE^+^ lung-derived and CFSE^–^ platelets were agonist stimulated, and platelet activation was quantified by flow cytometry for CD62P surface expression. Lung-derived platelets and BM-derived platelets responded similarly to thrombin and U46619 (*n* = 4 per group; results are representative of 2 independent experiments). (**E**) Isolated platelets from mice treated with CFSE o.p. were stimulated with PAM3CSK4 (PAM) (10, 50, or 100 μg/mL) or Tyrode’s or with loxoribine (Loxo) (250, 400, or 500 μg/mL) or 1:1 DMSO/H_2_O, and CD62P surface expression was compared in either CFSE^+^ or CFSE^–^ platelets (*n* = 4 per group; results are representative of 2 independent experiments). Data indicate the mean ± SEM. *P < 0.05, **P < 0.01, ***P < 0.001, and ****P < 0.0001, by (**D** and **E**) multiple *t* tests with Holm-Šidák multiple-comparison correction.

The literature has described a pathway from Flt3^–^ LT-HSCs directly to a Mk or MkP, particularly in the setting of emergency thrombopoiesis (24, 26, 28, 39, 42). This was recently shown to expand with age and associated with increased thrombosis (41). We therefore sought to assess the platelet production response of lung Mks during both basal and thrombocytopenic/inflammatory states. To quantify resident-lung Mk-derived platelets at steady state, we isolated platelets from mice given CFSE o.p. and CD42c-X649 antibody i.p. to label all lung-derived platelets (CFSE^+^) and platelets present on day 0 (X649^+^). Using flow cytometry, we selected CD41^+^ events and evaluated CFSE against day-0 CD42c-labeling antibody (Figure 3A). On day 1 after CFSE, the vast majority of CD42c^+^ platelets labeled on day 0 via i.p. injection were also CFSE^+^ (Figure 3A, middle plot), attributable to some leak of CFSE dye into the vasculature that did not reach tissue compartments. By post-CFSE day 5, we found a small portion of new (CD42c^–^), CFSE^+^ platelets and no longer saw meaningful numbers of the CD42c^+^CFSE^+^ platelet population (Figure 3A, right plot). The number of new CFSE^+^ platelets as a percentage of total platelets ranged from 1%–6% on days 3 and 5 after CFSE (Figure 3B). Because we did not have full labeling of all lung Mks (Figure 1B), we normalized CFSE^+^ platelets to CFSE^+^ Mks to represent the maximum potential contribution of lung-resident Mks to platelet production. This normalization suggests that approximately 10% of circulating platelets are lung-resident derived (Figure 3B, right panel). We used a complementary biotin-labeling o.p. approach to identify streptavidin binding–positive platelets derived from lung Mks. Like the CFSE model, approximately 5% of platelets were streptavidin^+^ on day 5 after biotin o.p. administration (Figure 3C). Taken together, these data indicated that at steady state approximately 5%–10% of circulating platelets come from lung-resident Mks.

Next, we assessed lung-derived platelet reactivity to common agonists. Platelets were stimulated with either thrombin (Figure 3D, left) or the thromboxane receptor agonist U46619 (Figure 3D, right), and platelet stimulation was measured using CD62P^+^ surface expression. Similar agonist responses occurred at every dose tested for both CFSE^–^ and CFSE^+^ platelets (Figure 3D). From these experiments, we conclude that lung-resident Mks produced approximately 10% of the platelet pool during homeostatic conditions, and these platelets reacted similarly to common agonists like thrombin and the thromboxane mimetic.

Platelets also activate in response to immune stimuli such as TLRs (43), and lung Mks have greater expression of TLRs compared with BM Mks. We compared the activation of CFSE^+^ lung-derived platelets to BM derived CFSE^-^ TLR responses. PAM_3_CSK4, a TLR1/2 agonist, increased CD62P expression on both CFSE^+^ lung-derived platelets and CFSE^–^ platelets (Figure 3E). At higher doses (50 and 100 μg/mL), CFSE^+^ lung-derived platelets exhibited a trend toward higher CD62P expression than that seen on CFSE^–^ platelets, although it was not statistically significant. The trend toward a greater response of CFSE^+^ lung-derived platelets to PAM_3_CSK4 also corresponded to greater TLR2 expression on CFSE^+^ lung-derived platelets (Supplemental Figure 3A). Loxoribine, a TLR7 agonist (44), also increased CD62P expression on CFSE^+^ lung-derived platelets and CFSE^–^ platelets, and at the highest dose (500 μg/mL), CFSE^+^ lung-derived platelets had higher CD62P expression than did CFSE^–^ platelets (Figure 3E). These data indicate that CFSE^+^ lung-derived platelets may be more sensitive to TLR agonist activation. CD62P expression can lead to platelet aggregation with leukocytes (43). Because CFSE^+^ lung-derived platelets exhibited greater CD62P expression in response to TLR agonists, we determined the amount of CFSE^+^ platelet leukocyte aggregates (PLAs). Circulating PLAs (CD45^+^CD42^+^) were enriched for CFSE^+^ platelets, with 30% of total PLAs being CFSE^+^, well above the 5% of CFSE^+^ circulating platelets (Supplemental Figure 3B). In contrast, CFSE^–^ PLAs comprised approximately 75% of the total, although total circulating platelets were 95% CFSE^–^ (Supplemental Figure 3B). These data highlight the idea that lung-derived platelets may be more “primed” to respond to immune stimuli.

### Lung-derived platelet production increases with acute and infection-associated thrombocytopenia.

**Figure 4:**
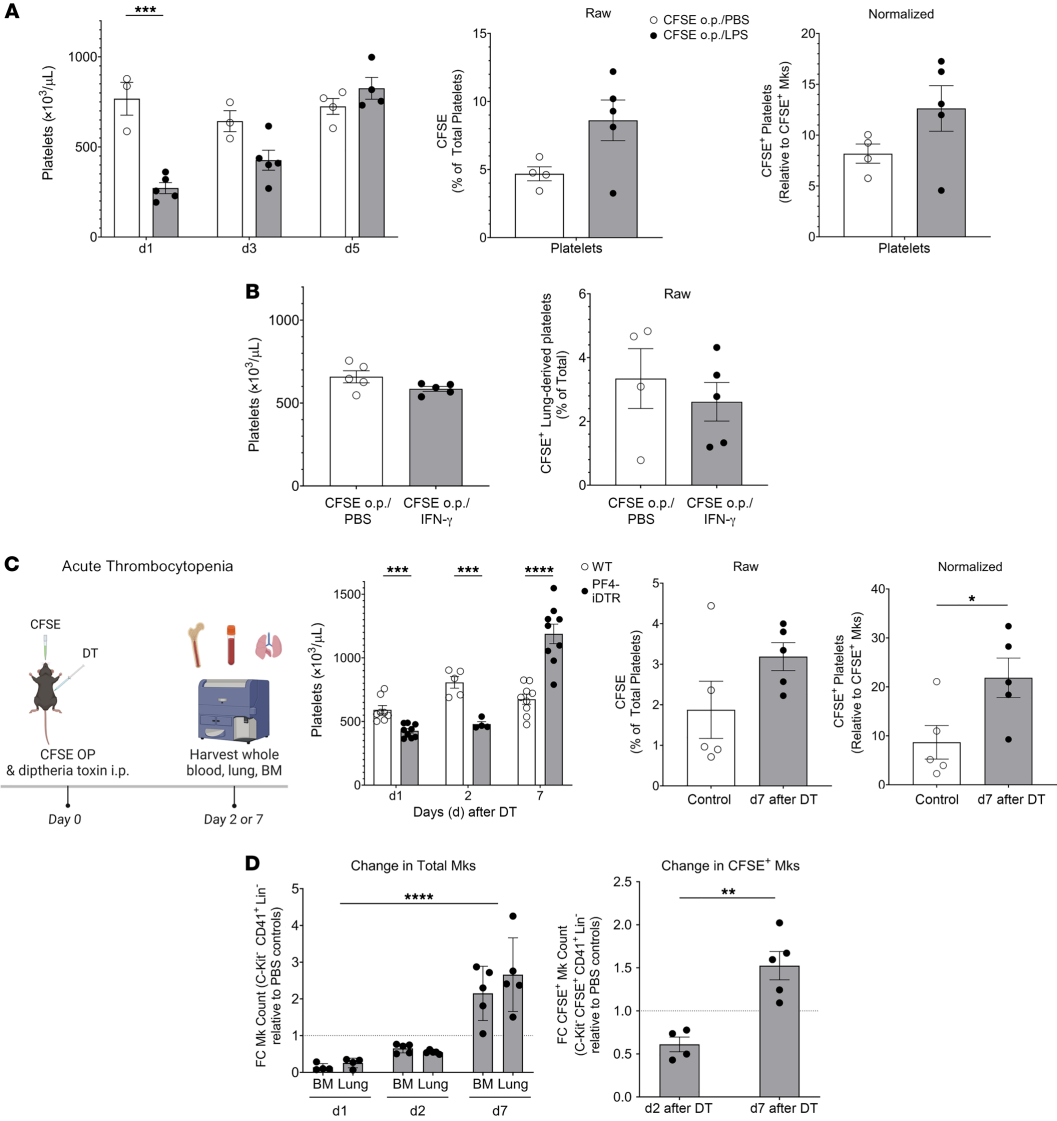
Lung-resident Mks respond to acute thrombocytopenia. (**A**) Mice were given CFSE o.p. and LPS or PBS i.p. LPS reduced platelet, but not Mk, counts and increased lung-derived platelets (*n* = 3–5 per group; results are representative of 2 independent experiments). (**B**) IFN-**γ** had no effect on platelet counts or CFSE^+^ platelets (*n* = 5 per group, representative shown from 2 independent experiments). (**C**) PF4^Cre^-iDTR mice treated with DT had reduced platelet counts (*n* = 5–9 per group from 2 independent experiments) and increased CFSE^+^ lung-derived platelets (*n* = 5 per group; results are representative of 2 independent experiments). DT treatment reduced BM and lung total Mks as well as CFSE^+^ Mks on day 2 after DT administration (*n* = 5 per group; results are representative of 2 independent experiments). (**D**) CFSE^+^ Mks relative to total Mks in control mice increased on day 7 after DT when Mk and platelet counts were recovered (*n* = 5 per group; results are representative of 2 independent experiments). Data indicate the mean ± SEM. *P < 0.05, **P < 0.01, ***P < 0.001, and ****P < 0.0001; (**A** and **C**) platelet count: multiple *t* tests with Holm-Šidák multiple-comparison correction; CFSE percentage and CFSE normalized: unpaired, 2-tailed *t* test; Mks: 2-way ANOVA with Tukey’s multiple-comparison correction; CFSE^+^ Mks: unpaired, 2-tailed *t* test; (**B**) unpaired, 2-tailed *t* tests; (**D**) Mks: 2-way ANOVA CFSE^+^ Mks: unpaired, 2-tailed *t* test.

Our prior studies, and those of others, demonstrated that lung Mks are immune differentiated (13–15). We therefore evaluated the role of lung Mk platelet production in inflammatory contexts by testing the effect of LPS. Mice were simultaneously given CFSE o.p. and LPS (1 mg/kg) or PBS i.p. Twenty-four hours later, platelet counts were reduced in the LPS-treated mice (Figure 4A, left). Three days later, CFSE^+^ lung-derived platelets as a percentage of total platelets, and normalized to CFSE^+^ Mks, were increased (*P* = 0.0597; Figure 4A, right panels). No changes in Mk numbers were observed in BM or lungs on day 3 after LPS treatment (Supplemental Figure 4A). To determine whether an inflammatory signal without associated thrombocytopenia increased lung Mk production, we treated mice with IFN-γ and observed no change in total or CFSE^+^ lung-derived platelets (Figure 4B), indicating that platelet production in the lung was not changed in the setting of immune stimuli without concomitant thrombocytopenia.

On the basis of these data, we sought to determine whether inflammation is required to induce increased lung-derived platelet production in an acute thrombocytopenia context. PF4-iDTR mice (45, 46) were administered diphtheria toxin (DT) to induce Mk apoptosis and acute thrombocytopenia for up to 5 days. On day 0, CFSE o.p. and DT i.p. were administered to WT control or PF4-iDTR mice. Whole blood, lungs, and BM were harvested at multiple time points (Figure 4C). Platelets rapidly declined on day 1 after DT and rebounded by day 7 (Figure 4C). CFSE^+^ lung-derived platelets increased on day 7 after DT (Figure 4C) and when normalized to the CFSE^+^ lung Mks, the proportion of lung-derived platelets doubled to 20% (Figure 4C). These data indicated that during acute thrombocytopenia, lung Mks increased their platelet production. DT-mediated depletion targets Mks, and we tracked the recovery of lung Mks. On days 1 and 2 after DT, total Mk numbers in both the BM and lung declined relative to those for controls and then rebounded by day 7 (Figure 4D). In contrast to some reports of PF4 being expressed in macrophage populations (47), we observed no decline in alveolar or interstitial macrophages on day 2 after DT, and CFSE^+^ Mks increased relative to controls (Figure 4D). Because CFSE remained only in the lung tissue, the increase in CFSE^+^ Mks on day 7 was from a lung source. These data indicated that thrombocytopenia led to an increase in lung-derived platelets.

To assess whether local progenitors may give rise to local CFSE^+^ Mks in the lungs, we evaluated lung and BM for changes in progenitors (CD150^+^ LKs) and MkPs (CD41^+^, CD150^+^ LKs) in DT-treated PF4-iDTR mice. We observed no change in the proportion of CD150^+^ LK progenitors in the lungs and no change in the percentage of CFSE labeling in this progenitor population in DT-treated mice (Supplemental Figure 4C). We also observed no change in the proportion of lung MkPs and no change in the percentage of CFSE labeling of the lung MkPs in DT-treated mice (Supplemental Figure 4C). These data suggest that the increase in CFSE^+^ Mks after acute depletion did not arise from a CFSE^+^CD150^+^ LKs or MkPs.

Because the acute depletion model demonstrated an increase in CFSE^+^ lung Mks, we attempted to evaluate the possibility of local proliferation of lung Mks. We evaluated the CFSE dye over time and found a decline in the percentage of Mks that were CFSE^+^ over an 8-week period in control mice (Supplemental Figure 4D). The MFI of CFSE^+^ Mks declined early and then was unchanged by day 56 (Supplemental Figure 4D). This may have been due to some lung Mk proliferation that led to platelet production and loss of those Mks, while other lung-resident Mks stayed “quiescent” during this period. Similarly, the percentage of lung LKs that were CFSE^+^ also declined (Supplemental Figure 4D), but the MFI of CFSE^+^ LKs did not meaningfully change (Supplemental Figure 4D).

Antibody-mediated platelet depletion was used as a complementary approach. We observed diminished platelet counts on day 1 following anti-Gp1bα antibody that recovered by day 5 (Supplemental Figure 4E, top left panel). However, we did not see an increase in CFSE^+^ platelets (Supplemental Figure 4E, top right panel). When the CFSE^+^ platelets were normalized to CFSE^+^ Mks, we also did not see an increase following antibody treatment (Supplemental Figure 4E, bottom right panel). The reduction in CFSE^+^ platelets following anti-Gp1bα antibody likely occurred as a result of a reduction in lung Mks, but not BM Mks (Supplemental Figure 4E, bottom left panel).

**Figure 5:**
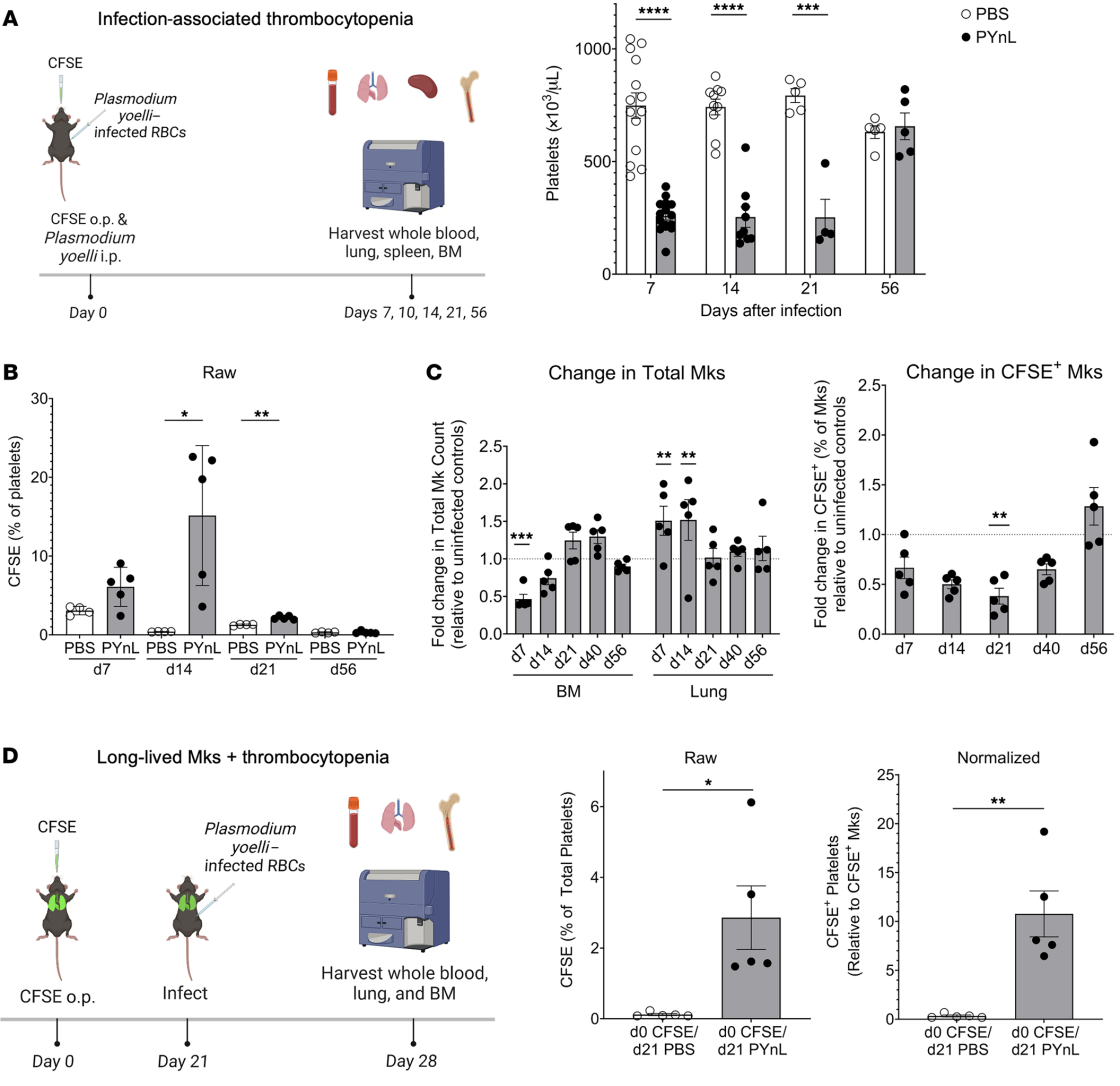
Lung-resident Mks respond to infection-associated thrombocytopenia. (**A**) Lung-resident, Mk-derived platelet counts were increased with PYnL infection–associated thrombocytopenia. Mice infected with PYnL had reduced platelet counts (*n* = 5–15 per group from 2 independent experiments) and (**B**) increased percentages of lung-derived CFSE^+^ platelets (*n* = 4–5 per group; results are representative of 2 independent experiments) in the first 2 weeks after infection. (**C**) Following PYnL infection, the number of BM Mks declined over the first 2 weeks, but the number of lung Mks increased (*n* = 4–5 per group per time point; results are representative of 2 independent experiments). The proportion of platelets that were CFSE^+^ was reduced early after infection, indicating an influx of new Mks from an extrapulmonary source. After infection recovery, the relative percentage of CFSE^+^ Mks in the lung were similar to that of control mice at the same time after CFSE. (**D**) Lung-resident Mks make platelets upon increased demand. Mice were treated with CFSE o.p. and infected 3 weeks later with PYnL. CFSE^+^ platelets were increased 1 week after infection (4 weeks after CFSE) (*n* = 5 per group; results are representative of 2 independent experiments). Data indicate the mean ± SEM. *P < 0.05, **P < 0.01, ***P < 0.001, and ****P < 0.0001; (**A**) platelets and (**B**) CFSE percentage of platelets and (**C**) CFSE^+^ Mks: multiple *t* tests with Holm-Šidák multiple-comparison correction; (**C**) total Mks: 2-way ANOVA with Holm-Šidák multiple-comparison correction; (**D**) CFSE percentage and normalized CFSE: unpaired, 2-tailed *t* test.

To test whether lung Mks increase platelet production in a disease context, we used a nonlethal murine malaria model of *Plasmodium yoelli* (PYnL) infection. PYnL results in thrombocytopenia and anemia from approximately day 7 to day 28 after infection, at which point the mice clear infection and recover (48). To track lung-derived platelets in infected mice, we gave CFSE o.p. and PYnL i.p. on day 0 and harvested blood and organs on day 7, day 14, and day 21 after infection, as well as on day 40 and day 56 when mice had cleared the infection (Figure 5A). We confirmed that the PYnL infection induced a sustained thrombocytopenia (Figure 5A). During the chronic thrombocytopenia phase of PYnL, we observed a large increase in circulating CFSE^+^ lung-derived platelets relative to uninfected controls, which normalized during the recovery time points on day 40 and day 56 (Figure 5B).

In evaluating the Mk compartments, on day 7 there was a decline in BM Mk numbers relative to those in uninfected controls (Figure 5C), however, on both day 7 and day 14, we noted an increase in lung Mks relative to uninfected controls (Figure 5C). In contrast to the increased splenic Mks observed with other infectious insults, such as in models of sepsis (49), Mks in the spleen declined throughout malaria infection and recovered by day 56 (Supplemental Figure 5). On day 14 we detected a decrease in the proportion of CFSE^+^ lung Mks relative to uninfected controls that normalized by week 8 after infection (Figure 5C, right panel). These data indicate that there was an early influx of lung Mks during infection that came from an extrapulmonary source. However, after the infection resolved, there was a relative increase in the proportion of lung Mks that were CFSE^+^, suggesting a local lung CFSE^+^ population as the Mk recovery source.

After infection recovery, very few CFSE^+^ platelets were noted, but CFSE^+^ Mks were still present in the lung. To test whether long-lived/resident lung Mks can be induced to make platelets in PYnL infection, we labeled lung Mks with CFSE dye, waited 3 weeks when approximately 15% CFSE^+^ Mks can be reliably found in the lung, and infected mice with PYnL (CFSE dye on day 0 and infected with PYnL on day 21; Figure 5D). One week after infection (day 28 after CFSE), CFSE^+^ circulating platelets increased in the infected group in comparison with uninfected controls (Figure 5D), indicating that resident Mks were recruited to make platelets with increased demand. When CFSE^+^ platelets were normalized to CFSE^+^ lung Mks, the infected group’s maximum lung-derived platelet production was approximately 10% of the circulating platelet pool (Figure 5D). These data demonstrate that resident Mks produced platelets when challenged with malaria-associated thrombocytopenia.

**Figure 6:**
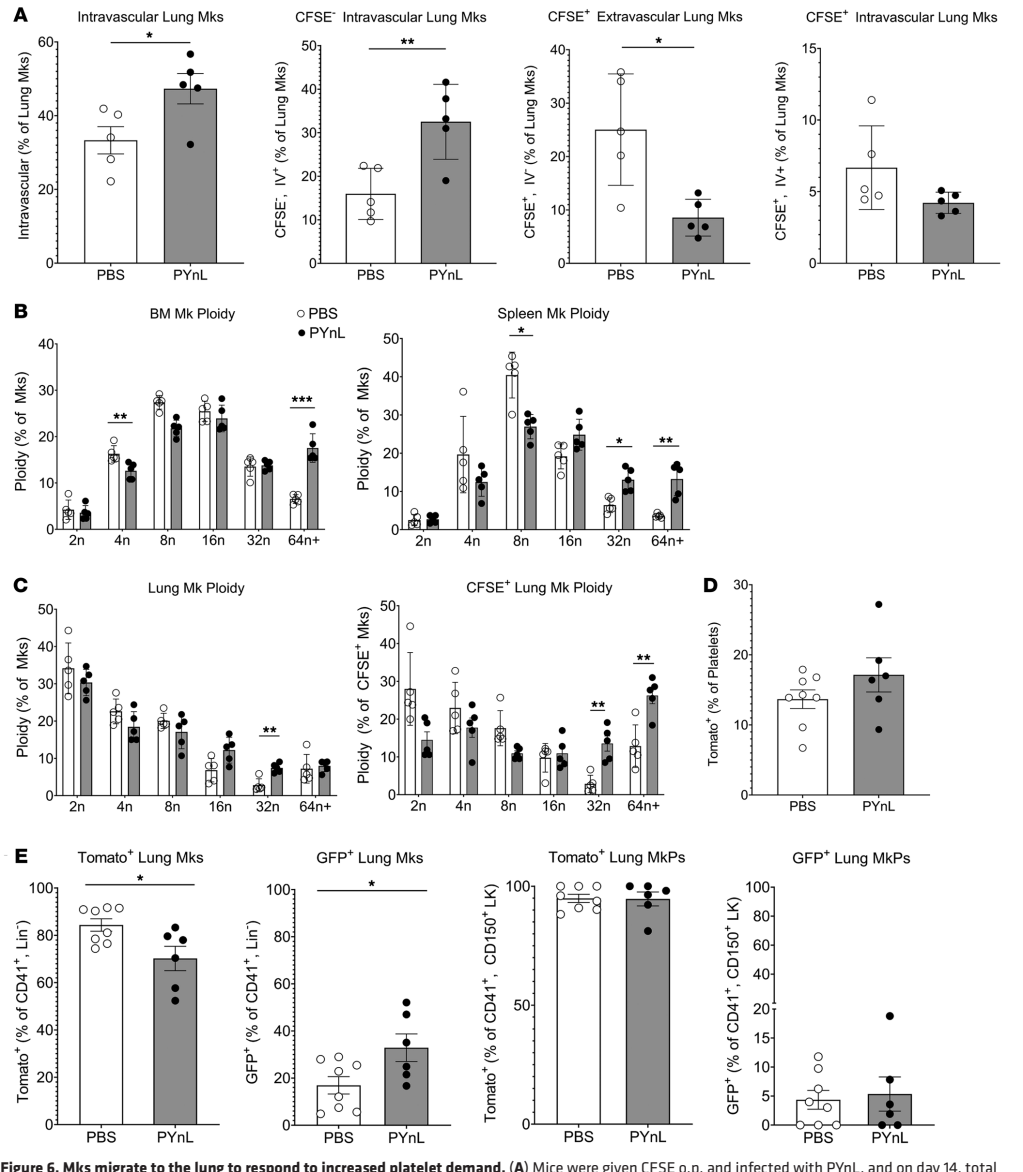
Mks migrate to the lung to respond to increased platelet demand. (**A**) Mice were given CFSE o.p. and infected with PYnL, and on day 14, total Mks and CFSE^+^ and CFSE^–^ intravascular (CD42d i.v. -positive) and extravascular (CD42d i.v. –negative) Mks were quantified. The increase in total intravascular Mks was driven by CFSE^–^ extrapulmonary Mks. On day 14, there was a decrease in CFSE^+^ extra- and intravascular Mks (*n* = 5 per group, representative shown from 2 independent experiments). (**B** and **C**) With PYnL infection, there was an increase in higher ploidy CFSE^+^ Mks in (**B**) BM and spleen as well as in (**C**) lung, including CFSE^+^ Mks (*n* = 5 per group, representative shown from 2 independent experiments). (**D**) FlkSwitch mice infected with PYnL had no significant change in Tomato^+^Flt3^–^ platelets on day 14 after infection, but (**E**) the percentage of lung Mks that were Tomato^+^Flt3^–^ slightly declined and that of GFP^+^Flt3^+^ Mks increased slightly compared with uninfected controls. There was no change in MkPs, indicating an influx of mature Mks from the BM that was largely Tomato^+^Flt3^–^ (*n* = 6–8 per group from 2 independent experiments). Data indicate the mean ± SEM. *P < 0.05, **P < 0.01, and ***P < 0.001, by unpaired, 2-tailed *t* test (**A**, **D**, and **E**) and multiple *t* tests with Holm-Šidák correction (**B** and **C**).

We also evaluated the ploidy and intravascular/extravascular status of lung Mks on day 14 of PYnL infection. We found an increase in the proportion of intravascular Mks relative to total lung Mks in PYnL-infected mice (Figure 6A, left). This increase was attributed to more CFSE^–^ intravascular Mks (Figure 6A), and we observed no change in CFSE^+^ intravascular Mks (Figure 6A). We also saw a decrease in CFSE^+^ extravascular Mks, but similar numbers of CFSE^+^ intravascular Mks at this time point (Figure 6A), as they had likely left the extravascular space to make platelets. On day 14 of PYnL infection, BM Mks had fewer 4n Mks and a larger number of 64n^+^ Mks (Figure 6B). In the spleen, lower ploidy states were depleted, particularly 8n Mks, but 32n and 64n^+^ Mks were increased (Figure 6B). In contrast, lung Mks remained largely 2n–4n, but there was an increase in 32n Mks on day 14 of PYnL treatment, and CFSE^+^ Mks showed a decline in 2n and 4n Mks at this time point (Figure 6C). These data indicate that the extrapulmonary influx of Mks was low ploidy, intravascular, and platelet producing.

Because there was an increase in extrapulmonary CFSE^–^ Mks during PYnL infection (Figure 4), we assessed whether Mks that migrate to the lung arise from Flt3^–^ progenitors. FlkSwitch mTmG mice were infected with PYnL, and tissue Mks were evaluated on day 14 after infection. We observed a slight, but not statistically significant, increase in Tomato^+^ platelets with infection (Figure 6D). The proportion of Tomato^+^Flt3^–^-derived Mks in the lung was slightly reduced, with a corresponding increase in the proportion of GFP^+^ lung Mks (Figure 6E). However, when we evaluated lung MkPs, we observed no change in the proportions of GFP^+^ or Tomato^+^ MkP populations (Figure 6E). These data further imply that increased lung Mks in PYnL infection arose from the BM. Despite the slight increase in GFP^+^ lung Mks at this time point, lung Mks were still predominantly Flt3^–^Tomato^+^, indicating that the Flt3^–^Tomato^+^ Mks have a preference for lung residence, but that during hematopoietic stress there may also be a movement of some low ploidy Mks derived from a Flt3^+^ differentiation pathway.

## Discussion

Our data indicate that at least a subpopulation of lung Mks were much longer lived than are BM Mks, originated via a Flt3-negative pathway from HSCs, and increased platelet production in response to thrombocytopenia. Previous literature assumed that a constant influx of Mks leave the BM, travel through the vasculature, and become “stuck” in the lung making platelets (50). In contrast, our parabiosis and lung Mk–labeling models demonstrated that in a 1-month period, a population of lung Mks were not replaced by a circulating source. Using CFSE and biotin labeling, we demonstrated that lung Mks could have a lifespan of up to 4 months, whereas BM Mks turned over within 1 week. The in vivo labeling methods validated in vitro studies showing that BM Mks live approximately 5 days (19). The mechanisms underpinning the prolonged lifespan of lung Mks remain unknown. Our data demonstrated an HSC-dependent, circulatory source for the replacement of lung Mks. However, at least some lung Mks remain quiescent or locally proliferate prior to their replacement. The majority of lung Mks are 2n, and perhaps the mechanisms that limit Mk polyploidization also confer their quiescent, yet proliferative, potential. Insights into Mk lifespan may be important for improving current strategies to produce platelets ex vivo and will provide important context for studying Mk immune differentiation. It is also important to note that our studies were completed in mice and provide a foundation for future human-based studies.

Mounting evidence supports the concept that there is an alternative, direct path from an HSC to Mk that bypasses progenitor stages (24, 25, 28, 39). Here, we showed that lung Mks predominantly arose through a direct pathway from a Flt3^–^ HSC. In contrast, few BM and splenic Mks (<10%) potentially arose from a direct pathway in baseline conditions at the relatively young ages we studied. The apparent bias of Flt3^–^ Mks for lung tissue could be due to better fitness of those Mks for the lung environment or perhaps Flt3^–^ Mks have migratory potential and are more likely to leave the BM, enter the lung, and have a longer lifespan. The distinct differentiation of lung Mks may also encode their immune-modulatory phenotype that may expand with age (41). A recent study used a CD48-dependent reporter to track canonical versus “direct” pathways of Mk differentiation and found an approximately equal distribution in the lung (51). It is important to note that CD48 is weakly expressed by HSCs and MPPs, and its expression is increased in restricted progenitors (51). The FlkSwitch mouse labels cells beginning at the MPP stage of differentiation, so the timing in the initiation of the reporter expression may be an important difference in these mouse model findings. These 2 mouse models may also highlight potential mechanisms of Mk differentiation in the BM that bias Mks for the lung environment, which need more investigation.

Our methods indicated that lung-resident, Mk-derived platelet production was approximately 10% of circulating platelets, which is lower than some previous estimates (13, 16). The methods we developed label the lung-resident Mks that are largely extravascular, and we cannot rule out a constant migratory Mk source that does not leave the vasculature, but contributes to lung-derived platelet production in a more transient manner. We also determined that lung-resident Mks contributed more platelets in the settings of both acute and infection-associated thrombocytopenia. The generation of lung Mks via a direct pathway from HSCs would enable lung Mks to serve as a reservoir for platelet production that rapidly responds to thrombocytopenia. Of note, anti-GPIb antibody–mediated platelet reduction, did not induce more lung Mk–derived platelets. While we note this may be due to the reduction of lung Mks more than of BM Mks, our past work showed that lung Mks express more Fc receptors and are phagocytic (14), so we cannot rule out an antibody-mediated mechanism that may also be relevant in immune-mediated thrombocytopenia. Another potential limitation to note is that BM Mks are straightforward to isolate, while lung Mks require digestion of a complex and extracellular matrix–rich tissue. Therefore, if higher ploidy Mks are more fragile to digestion we may be underestimating their numbers and ploidy in lung tissue.

This work highlights the distinct biology of lung Mks and describes their platelet contribution, particularly during thrombocytopenic states. It remains to be explored if lung Mks generate more immune-differentiated platelets in diverse disease contexts. Further research is required to better characterize the role of Mks produced via the direct/alternate pathway in all tissue environments. These data add to a growing body of evidence demonstrating the relevance of lung Mks to health and disease.

## Methods

### Sex as a biological variable.

Both male and female mice were used for all experiments conducted using WT, PF4^iDTR^, and MDS1^CreERT2^ mice. For experiments conducted using FlkSwitch-mTmG mice, only male mice were used, as the FlkSwitch transgene is on the Y-chromosome (22), making Flt3-dependent lineage tracing impossible in female mice.

### Mice.

All mice used were on a C57/Bl6 background. Mice not bred in-house were purchased from The Jackson Laboratory or obtained from other collaborators as described below. PF4^iDTR^ mice (45) were used for Mk/platelet depletion studies. For depletion, 400 ng DT (MilliporeSigma, D0564-1MG) in sterile water (16 μg/kg) was administered to mice in a 100 μL i.p. injection.

MDS1^CreERT2^ mice were bred with Rosa26-tdTomato mice as previously described (37). Tamoxifen was given a total of 6 times over a 2-week period by i.p. injection to induce the fluorescent reporter. Tamoxifen (37.5 mg/kg) was prepared as described previously (52). FlkSwitch mice were obtained from Anna Beaudin (University of Utah, Salt Lake City, USA) and have been previously described (22, 40).

### CFSE dye by o.p. administration.

CFSE is a fixable, cell-permeable dye that allows for long-term labeling of cells. CFSE stock (25 mM, Thermo Scientific, C1157) was diluted with Iscove’s Media to 8 mM and administered to mice via the o.p. route. Briefly, mice were anesthetized with isoflurane and hung by their teeth on dental floss. The tongue was gently pulled to prevent swallowing. Thirty microliters of 8 mM CFSE was pipetted directly into their oropharyngeal cavity, and mice remained on the dental floss for 30–45 seconds to allow for aspiration of the CFSE dye. Organs (bone marrow, spleen, and lung) and blood were collected to assess for the presence of CFSE at all time points analyzed. For platelet analyses, CFSE dye was given o.p., and platelet labeling antibody (Emfret X649) was given via the i.v. route simultaneously. For normalization calculations measuring the overall contribution of lung Mks to lung platelets, the following calculation was made:

(Equation 1)
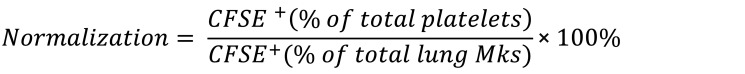


### Biotin administration.

EZ-link Sulfo-NHS-SS-Biotin (Thermo Fisher Scientific, 20217) was prepared at 5 mg/mL concentration. Biotin was administered either via the o.p. route in 30 μL volume (as described above) or i.v. as previously described (34). PE-streptavidin (BioLegend) or BV711-streptavidin (BioLegend) was used for the detection of in vivo biotin labeling.

### Blood collection, complete blood counts, and platelet isolation and activation.

Mice were bled via the retro-orbital route into EDTA tubes (Thermo Fisher Scientific, NC9990563). Complete blood counts (CBCs) were performed using Abaxis VetScan HM5. Platelets were collected via the retro-orbital route into heparanized Tyrode’s and isolated as described previously (53). For activation, isolated platelets were incubated with thrombin (0, 0.2, 0.5, or 1 U) or U46619 (0, 2, 5, or 10 U) for 10 minutes.

### Immune stimuli.

For immune stimuli studies, LPS or IFN-γ were administered i.p. to mice. LPS was given at 1 mg/kg as a 1-time dose (MilliporeSigma, L6259). Recombinant mouse IFN-γ (R&D Systems, 485MI100/CF) was administered once daily i.p. at 0.04 mg/kg for a total of 3 consecutive days.

Plasmodium yoelli (PYnL) was used as a nonlethal chronic infection model of thrombocytopenia. Mice were infected with 500,000 infected RBCs via i.p. injection and monitored on post-infection days 7, 10, 14, 21, 28 and 56. For the fold change of total Mk counts and CFSE^+^ Mk counts, the following equations were used: 

(Equation 2)
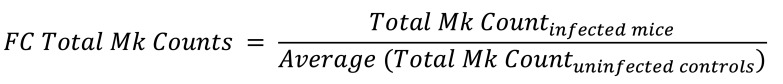


(Equation 3)
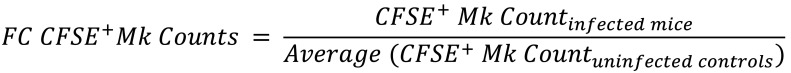


### Parabiosis.

Parabiosis surgeries were performed, with postoperative monitoring as described previously (54, 55). In brief, 8-week-old female mice were dosed with CFSE o.p. 7–10 days prior to surgery. Preoperative carprofen (5 mg/kg), extended-release buprenorphine (1 mg/kg), and enrofloxacin (2.5 mg/kg) were administered s.c. Anesthetized WT and mTmG mice were shaved to remove a wide margin of fur at the level of the flank at the intended surgical site. During surgery, mice received matched longitudinal skin incisions from 0.5 cm above the olecranon to 0.5 cm below the knee joint. Subcutaneous tissue was bluntly dissected to expose the olecranon and knee joints of each mouse. A suture was passed around each olecranon and secured with 4 throws of a square knot. This procedure was repeated at the knee joints. The skin suture was closed using 5-0 Vicryl interrupted mattress sutures. Interrupted reinforcement sutures were also used. Postoperative monitoring was performed at least twice daily. Multimodal analgesia was continued for at least 3 days postoperatively, with a supplemental soft diet and subcutaneous fluids provided as needed. Analyses of tissues were performed 4 weeks after surgery.

### Single-cell suspensions for flow cytometry, ImageStream flow cytometry, and FACS.

For bone marrow single-cell suspensions, 1 tibia and 1 femur were harvested and flushed with isolation buffer using a 20 gauge needle. Isolation buffer consisted of 1 mM EDTA and 2.5% FBS in PBS. Whole lungs were dissected and placed into collagenase type 2 digestion buffer as described previously (17). All cells were filtered with a 100 μm filter and used for downstream analyses. For flow sorting, single-cell suspensions were ACK lysed and washed with PBS prior to staining.

### Flow cytometry and ImageStream reagents.

Antibodies against the following proteins were used: CD41 (MWReg30, BioLegend), Ter119 (Ter119, BioLegend), CD19 (MB19-1, Thermo Fisher Scientific), CD3 (17A2, BioLegend), CD11b (M1/70, BioLegend), Gr-1 (RB6-8C5), c-Kit (ACK2, BioLegend), GP1bβ (Emfret, X649), PE-streptavidin (BioLegend), BV711-streptavidin (BioLegend), CD62P (RMP-1, BioLegend), Sca1 (D7, Thermo Fisher Scientific), Flt-3 (A2F10, Thermo Fisher Scientific), CD48 (HM48-1, BioLegend), and CD150 (TC15-12F12.2, BioLegend).

### Statistics.

All statistical tests were performed using GraphPad Prism, version 8. The appropriate statistical test was performed according to the experimental design. For comparison of 2 groups, a 2-tailed *t* test was used assuming similar SDs between groups. For comparisons of multiple groups across time, a 2-way ANOVA with Tukey’s multiple-comparison test was used. For comparisons of multiple groups at 1 time point, an ordinary 1-way ANOVA was used with multiple comparisons (corrected with the Šidák method). For comparisons of control and treated groups across organs, multiple *t* tests with correction for multiple comparison (Holm-Šidák method) were used. A *P* value of less than 0.05 was considered significant. In bar graphs, individual points represent individual replicates from an experiment. Data were replicated in separate independent experiments. All data represent the mean ± SEM.

### Study approval.

All vertebrate animal studies were approved by the University of Rochester Animal Care and Use Committee under protocol no. 2009-022E.

### Data availability and sharing.

Data are available upon reasonable request to the corresponding authors. All data for individual replicates of experiments are provided in the Supporting Data Values file.

## Author contributions

ACL designed studies, performed experiments, acquired data, analyzed data, and wrote the manuscript. KEG designed studies, performed experiments, acquired data, and analyzed data. MWM, CL, SKT, PDK, ADK, LAV, KEN, DM, GEM, and PM all performed experiments and acquired data. JP designed studies, analyzed data, provided reagents, and wrote the manuscript. CNM designed studies analyzed data, provided reagents, and wrote the manuscript.

## Supplementary Material

Supplemental data

Supporting data values

## Figures and Tables

**Table 1:**
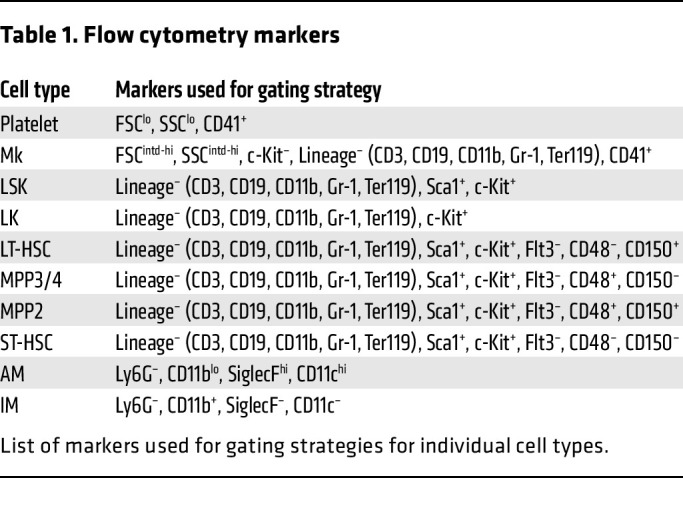
Flow cytometry markers

## Version 1 09/20/2024

In-Press Preview

## Version 2 11/15/2024

Electronic publication
