# Supplementary material for: Long-lived lung megakaryocytes contribute to platelet recovery in thrombocytopenia models
Source: J Clin Invest. 2024 Sep 19;134(22):e181111. doi: 10.1172/JCI181111 (PMC11563682; doi:10.1172/JCI181111)
Supplement: Supplemental data [file jci-134-181111-s246.pdf]

Supplemental Figures

Supplementary Figure 1

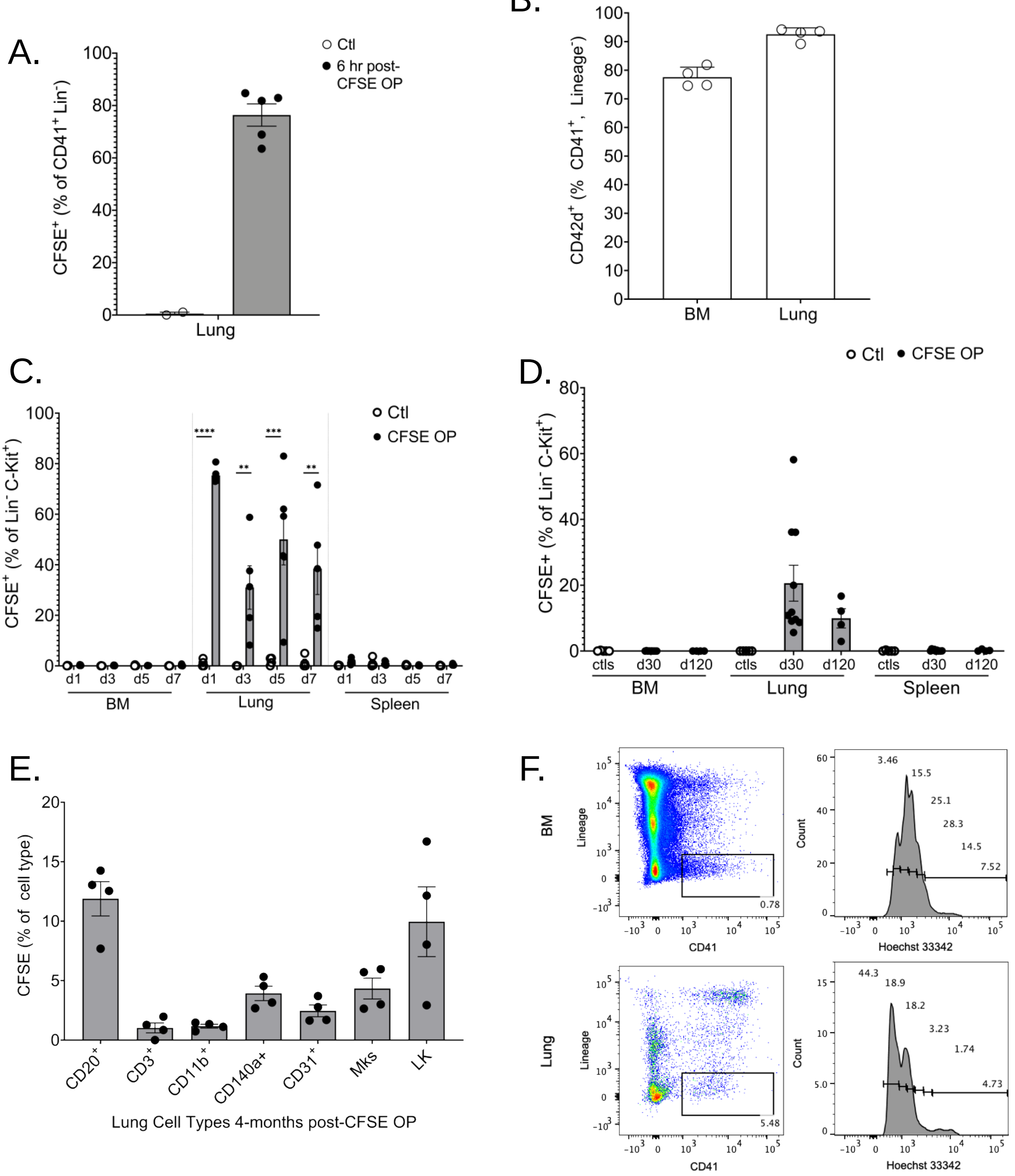

G.

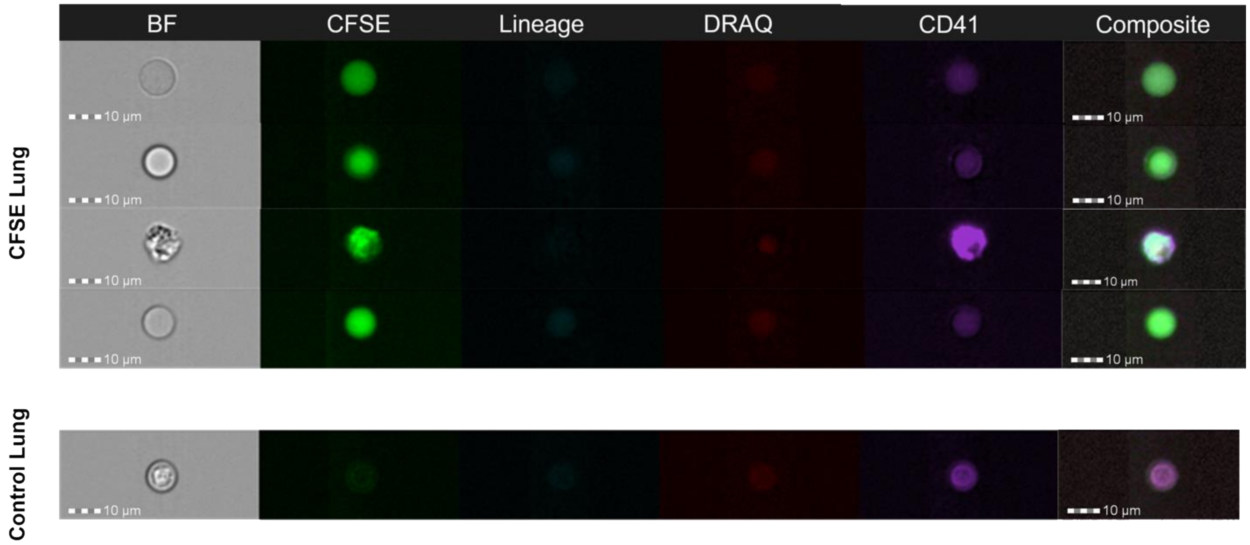

Supplemental Figure 1. A) CFSE labeling six hours post administration in CFSE or control mice. B) CD42d labeling of CD41^{+}, Lineage^{-} cells from lung and BM using flow cytometry. C) CFSE oropharyngeal (OP) dye labeled Lineage^{-} c-kit^{+} cells (LK) from d1-d7 (Two-way ANOVA with Tukey's Multiple Comparison Tests). D) CFSE OP dye labeled LK cells d30 or d120 later (Multiple *t*-tests with Holm-Sidak multiple comparison correction test). E) CFSE labeling of multiple cell types 4 months post-CFSE. F) Representative flow cytometry gating for CD41^{+}, Lineage^{-} cells and Hoescht 33342 staining for ploidy gates are shown (2N, 4N, 8N, 16N, ≥32N from left to right). G. Representative imaging flow cytometry of lung CFSE dye in CD41^{+}, Lineage^{-} cells from CFSE-OP treated mice (first four rows represent separate mice) or control mouse (bottom row).

Supplementary Figure 2

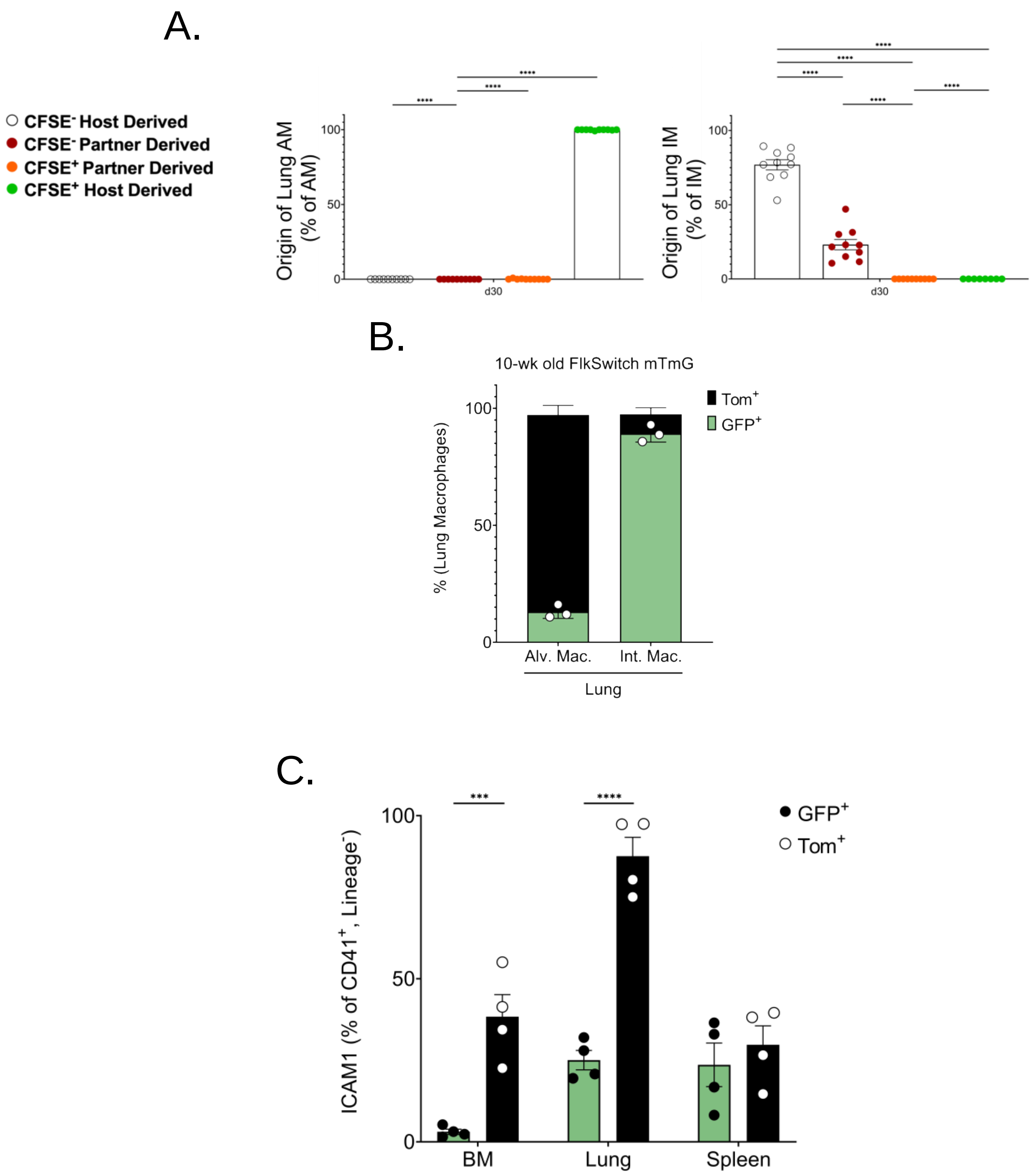

Supplemental Figure 2. A) One month after parabiosis surgery, all AMs remained CFSE$^+$, host-derived and IMs were replaced by CFSE$^-$ host-derived cells with 23% from the CFSE$^-$ partner, indicating circulatory replacement of IMs from the partner parabiont. No IMs were CFSE$^+$ from the partner (orange) or host (green). B) GFP and Tomato expressing alveolar and interstitial macrophages as % of lung macrophages in 10-week-old FlkSwitch mTmG mice. C) ICAM1 expression on CD41$^+$, Lineage$^-$ GFP or Tomato cells in in BM, lung and spleen. (Two-way ANOVA with Sidak multiple comparison test correction shown for B and C).

Supplementary Figure 3

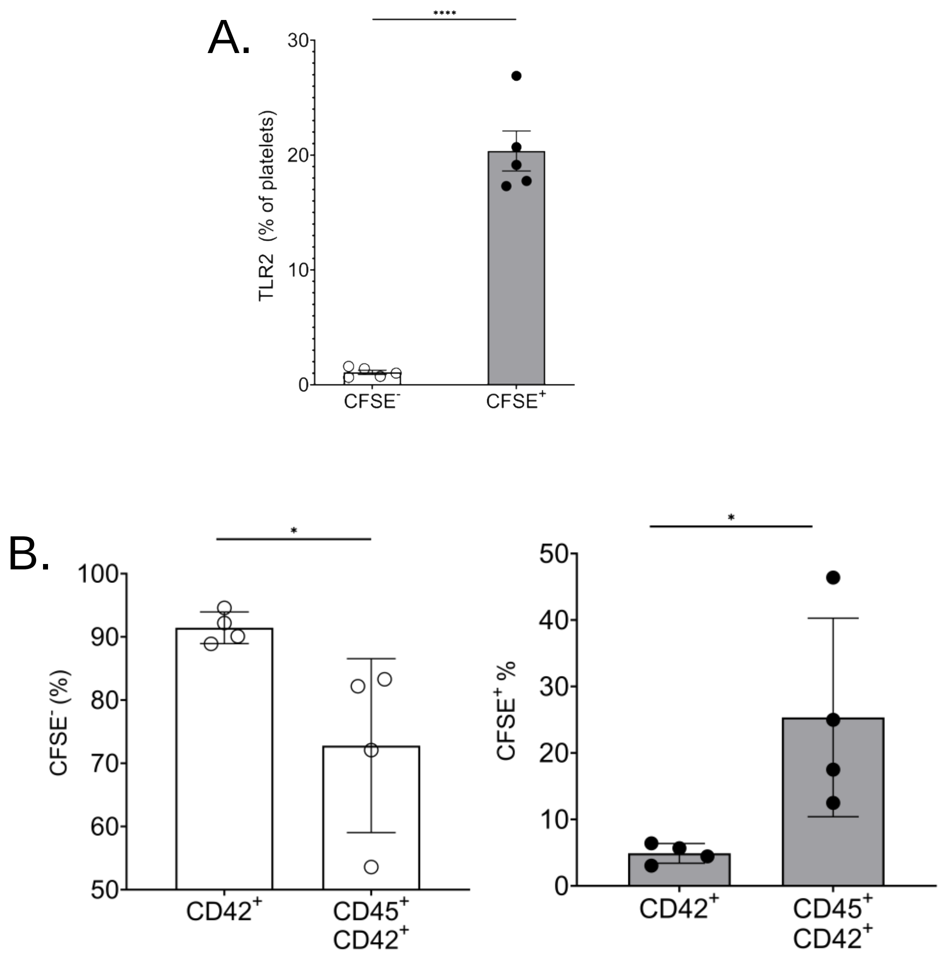

Supplemental Figure 3. A) Platelet TLR2 expression measured using flow cytometry in CFSE$^+$ or CFSE$^-$ platelets. B) Percent CFSE$^-$ (left panel) or percent CFSE$^+$ (right panel) of CD42$^+$ platelets or CD45$^+$ CD42$^+$ platelet leukocyte aggregates in peripheral blood (unpaired, two-tailed *t*-tests shown for A and B).

Supplementary Figure 4

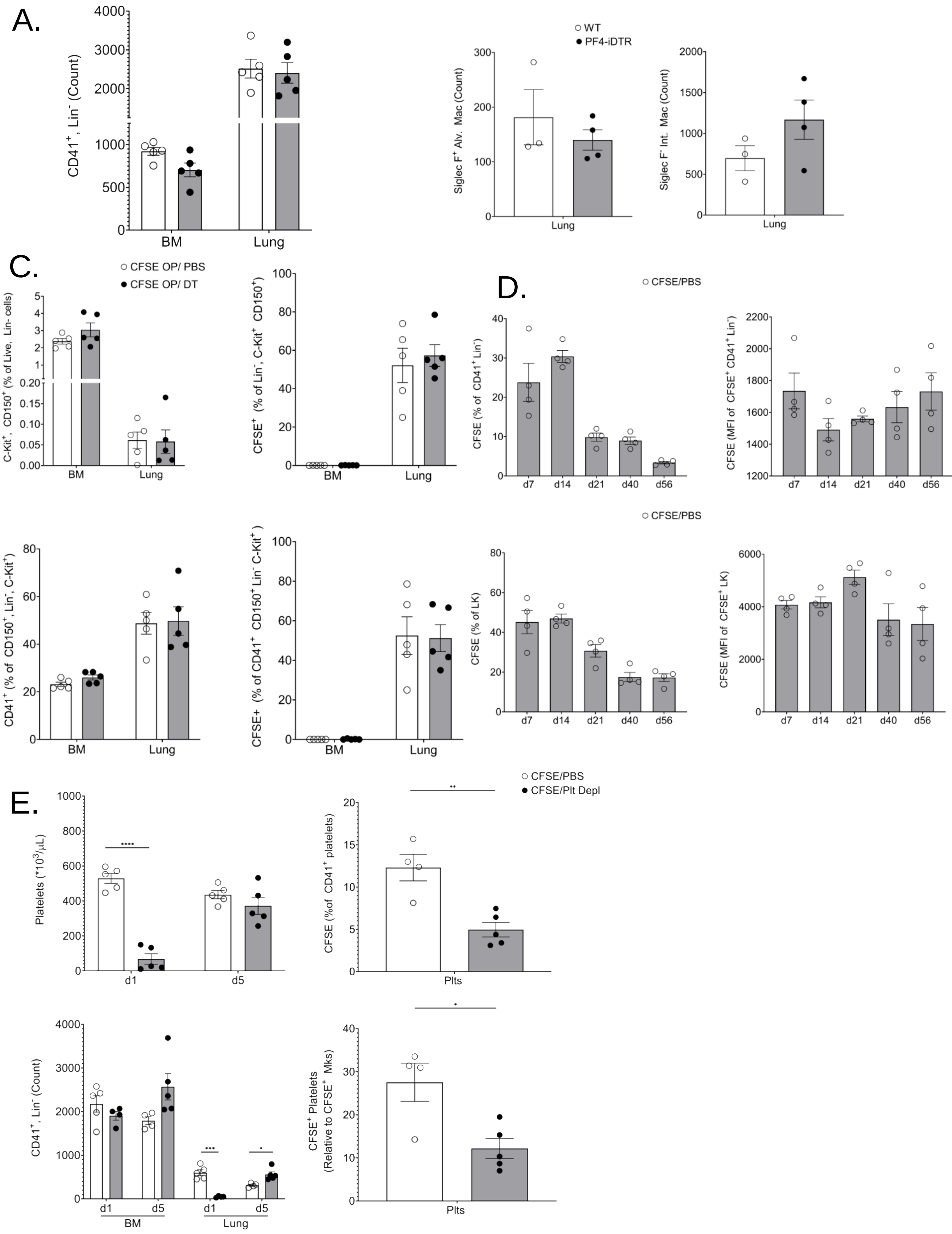

Supplemental Figure 4. A) Numbers of BM and Lung Mks on d3 post-LPS. B) Numbers of Siglec-F^+ alveolar lung macrophages (Alv. Mac) in left panel and Siglec-F^- interstitial lung macrophages on right panel in control and DT-treated PF4-iDTR mice on d2. C) C-Kit $^+$, $CD150^+$, Lin^- cells (upper left) and their CFSE % (upper right) and $CD41^+$ $CD150^+$ LK (bottom left) and their CFSE % (bottom right) in control and DT-treated iDTR mice. D) The change in $CFSE^+$ % (upper left) and MFI (upper right) of $CD41^+$ $Lineage^-$ cells and LK d1-d56 (bottom left and right) in the lung. E) Platelet counts on d1 and d5 in control or platelet-depleted (Ab-mediated) mice (upper left panel); new $CFSE^+$ platelets percent of total platelets (upper right panel); new $CFSE^+$ platelets normalized to lung $CFSE^+$ Mks (bottom right panel); $CD41^+$, $Lineage^-$ numbers (bottom left panel).

Supplementary Figure 5

F.

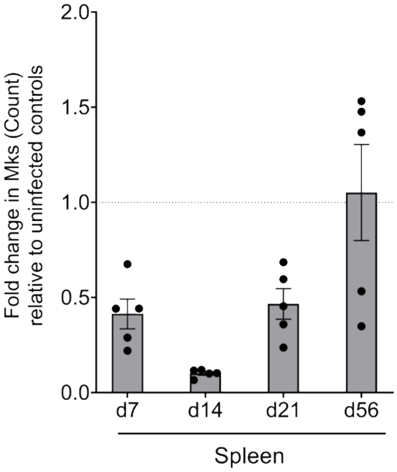

Supplemental Figure 5. Fold change in spleen Mks relative to uninfected controls on d7, 14, 21, and 56 post-infection with PYnL.
